# Biased and unbiased perceptual decision-making on vocal emotions

**DOI:** 10.1038/s41598-017-16594-w

**Published:** 2017-11-24

**Authors:** Mihai Dricu, Leonardo Ceravolo, Didier Grandjean, Sascha Frühholz

**Affiliations:** 1 Swiss Center for Affective Sciences, Campus Biotech, University of Geneva, 1202 Geneva, Switzerland; 2 Department of Experimental Psychology and Neuropsychology, University of Bern, 3012 Bern, Switzerland; 3 Department of Psychology and Educational Sciences, University of Geneva, 1205 Geneva, Switzerland; 4 Department of Psychology, University of Zurich, 8050 Zurich, Switzerland; 5 Neuroscience Center Zurich, University of Zurich and ETH Zurich, Zurich, Switzerland; 6 Center for Integrative Human Physiology (ZIHP), University of Zurich, Zurich, Switzerland

## Abstract

Perceptual decision-making on emotions involves gathering sensory information about the affective state of another person and forming a decision on the likelihood of a particular state. These perceptual decisions can be of varying complexity as determined by different contexts. We used functional magnetic resonance imaging and a region of interest approach to investigate the brain activation and functional connectivity behind two forms of perceptual decision-making. More complex unbiased decisions on affective voices recruited an extended bilateral network consisting of the posterior inferior frontal cortex, the orbitofrontal cortex, the amygdala, and voice-sensitive areas in the auditory cortex. Less complex biased decisions on affective voices distinctly recruited the right mid inferior frontal cortex, pointing to a functional distinction in this region following decisional requirements. Furthermore, task-induced neural connectivity revealed stronger connections between these frontal, auditory, and limbic regions during unbiased relative to biased decision-making on affective voices. Together, the data shows that different types of perceptual decision-making on auditory emotions have distinct patterns of activations and functional coupling that follow the decisional strategies and cognitive mechanisms involved during these perceptual decisions.

## Introduction

Every day we consistently discriminate, identify and evaluate the emotions of others, that is, we make decisions about which emotions we perceive. Decision-making on perceived emotions involves gathering information about the emotional state of another person through the senses, evaluating and integrating that information according to the goals^1^ and predictions^2^ of the perceiver, and forming a decision. In contrast to decision-making in general, perceptual decision-making (PDM) emphasizes the role of available sensory information in choosing one option from a set of alternatives^3^. In experimental paradigms, the perceptual decision-making process is investigated with an additional step in the form of a behavioral response (e.g. a verbal report or a button press).

In the classic framework of information processing, PDM should be the product of a succession of steps from sensory processing to decision formation and, if necessary, motor execution^4^. However, attempts to fit this division of labor to neural data have encountered several challenges. Functions that should be unified appear distributed throughout the brain^5^, whereas functions that should be distinct appear to involve the same regions^6^, or even the same brain cells^7,8^. Traditionally, extracting sensory information about the world has been shown to take place in specific regions in the brain (e.g. tactile information^9^; faces^10^, voices^11^). The intermediate stage of PDM, i.e. decision formation, is arguably the most enigmatic due to the unsuccessful attempts of researchers to pinpoint specific anatomical regions. A major source of this elusiveness is the varying definition of what should implicate a region in decision formation, and the subsequent methodology used^12,13^. As a result, there are broad inconsistencies in both the number and the location of brain regions implicated in perceptual decision formation in humans^14–20^.

Despite the ongoing debate, the general principle that arises from the literature on PDM is the need for strong bilateral connections between regions of sensory encoding, decision formation and, if required, regions of motor execution. Because there are separate pathways for processing stimuli depending on their perceptual modality and their inherent features (e.g. the ‘what’ and the ‘where’ pathways^21–23^), it stands to reason that the brain networks involved in PDM might also be modulated by these factors. Furthermore, top-down signals from prefrontal cortices to sensory-processing regions ensure that perception remains flexible, in line with the task demands and contextual needs^24–26^. Consequently, instead of a single region or network involved in perceptual choices, likely several networks and mechanisms are involved in deciding what is being perceived. Our brains are factoring in the sensory information at hand as well as contextual cues and prior experience with the stimulus^27–29^, and decide what is being perceived.

There is an impressive amount of information both in the form of empirical data and computational models on how PDM occurs in the visual domain^13,30^. Significantly less literature exists for the auditory domain, where the decisional process is gradually constrained by serially incoming information. Nevertheless, the same basic principles are assumed in auditory PDM^17^. For perceiving human voices, in particular, the superior temporal cortex (STC) is a region of sensory encoding^11,31^ while the inferior frontal cortex (IFC) is likely a region of decision formation^32–34^. The IFC is the receiving end of both dorsal and ventral processing streams with information fed from the STC^23^. In line with the notion that auditory regions parse the speech signal at different time scales^35–37^, the IFC may have access to several types of temporal acoustic information. The IFC is highly convoluted, cytoarchitectonically diverse and asymmetric not only on the whole^38^ but also at the level of its sub-regions^39–42^. In effect, this makes the IFC a gyral complex. Aside from structure and morphology, its sub-regions are also functionally distinct^43,44^ yet highly interconnected^45^. The sub-regions may further serve distinct mental functions, with the left pars opercularis having as many as five separate functional clusters, as revealed by connectivity-based parcellations^44^.

Specifically concerning decisions on emotional voices, the inferior frontal cortex (IFC) and the orbitofrontal cortex (OFC) have consistently been involved in evaluating perceived vocal emotions^36,46^. Consequently, several models have placed the IFC and OFC in a crucial role for evaluating affective voices^47,48^. These structures have close functional and structural connections with the auditory cortex^23,49^, especially during more cognitively demanding decisions^50^, and they display functional specialization in processing affective voices at the level of their sub-regions, depending on the attentional focus and the medium of vocal expressions^36^. To process emotions from the human voice, a temporo-frontal network is believed to mostly operate in a hierarchical manner^47,51^, including sensory-feature extraction and integrative processes in the superior temporal cortex (STC), as well as evaluative decisions in the IFC/OFC. Additional subcortical regions, such as the amygdala, are recruited at both sensory processing and decision-making levels^52,53^. Thus, PDM on affective voices consists of a core network of auditory, inferior frontal, and subcortical brain regions^54^.

**Figure 1:**
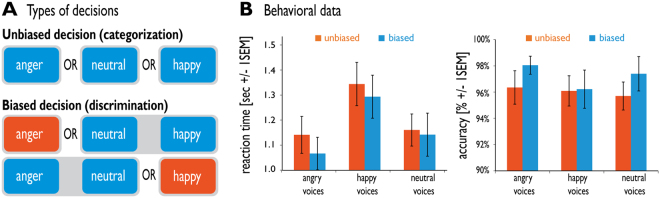
Task instructions and behavioral performance. (**A**) During unbiased decisions (“A or B or C” decision; upper panel), participants were asked to categorize affective voices as angry, neutral, or happy. During biased decisions (“A or not-A” decision; lower panel), participants were asked to decide whether a perceived voice was either angry or not angry, or happy or not happy. The color points to the presence (red color) or absence (blue color) of an a priori bias towards the choice options. **(B)** Reaction times and accuracy for biased and unbiased decisions on angry, happy, and neutral voices.

The present study used two frequent types of PDM and investigated how the brain adapts to contextual demands in determining what is being perceived. In separate blocks, we asked participants to discriminate identical emotional voices based on two sets of instructions that emphasized the presence or absence of an a priori bias (Fig. 1A). An *unbiased decision* assumes an equal race between several competing choices, and the final answer is decided in a winner-takes-all fashion after enough information has been serially accumulated^55^. Such decisions usually appear in contexts where the perceiver has sufficient time to make an informed decision according to several choice options. For decisions with three possible choices, this can be represented as an “A-or-B-or-C” decision task. A *biased decision*, on the other hand, channels the decisional process towards a particular target (i.e. a specific emotion), and the perceiver tries to confirm or disconfirm the match between a perceptual template of the target answer and the external stimulus^56^. Regardless of the number of possible choice options, this decision can be represented as an “A-or-not-A” decision, such as selectively monitoring for threat information in a dangerous environment (i.e. “threat” or “no threat”). Biased decisions are a case of conditional expectations, in which the anticipation of one perceptual alternative is favored^56^. As such, they go beyond a classic two-alternative forced-choice tasks by making the target option (e.g. a threat information) more salient^57–59^, channeling cognitive resources towards the target characteristics and away from unwanted or irrelevant features^30,60^, and improving speed and accuracy of decisions^61^.

To reveal patterns of brain activation and functional connectivity for biased and unbiased decisions on affective voices, we used functional magnetic resonance imaging (fMRI) and psychophysiological interaction (PPI) analysis. Based on previous literature^23,36,49,50,62,63^, we focused specifically on the temporo-frontal network and the amygdala. We hypothesized that biased and unbiased decisions would recruit distinct sub-regions within the IFC/OFC, and that unbiased decisions would display stronger functional coupling between frontal, auditory, and limbic regions relative to biased decisions. A further hypothesis was that, if biased and unbiased decisions posed different levels of difficulty to the participants (i.e. maintaining three choice options instead of two choice options in working memory, respectively), then we would expect to see differential practice effects on reaction times and accuracy from the first block to the second for unbiased decisions but not biased decisions.

## Results

### Behavioral analysis

For all behavioral analyses, condition-specific mean reaction times were computed for each participant. To determine whether there are differential practice effects for biased and unbiased decisions, we included block order as an additional factor in our behavioral analyses. A two (*decision*: biased and unbiased) by three (*emotion*: angry, happy, neutral) by two (*block order*: first and second) repeated-measures ANOVA on reaction times during correct trials revealed a main effect of decision (F_1,15_ = 6.228, p = 0.025) in which biased decisions were performed faster than unbiased decisions, and a main effect of emotion (F_2,30_ = 36.828, p < 0.001), but no interaction effect between decision and emotion (F_2,30_ = 1.398, p = 0.263). Pairwise comparisons (Bonferroni correction) revealed that there were no differences between making decisions on angry versus neutral voices (p = 0.186), whereas happy voices were processed more slowly than both neutral voices (p < 0.001) and angry voices (p < 0.001) (Fig. 1B). In addition, we found no main effect of block order on reaction times (F_1,15_ = 2.232, p = 0.156), nor an interaction effect between block order and decisional strategy (F_1,15_ = 0.06, p = 0.940) or block order and emotion (F_1,15_ = 0.071, p = 0.931) or decision x emotion x order (F_2,30_ = 0.416, p = 0.663).

The overall accuracy averaged across decisions and participants was very high (96.64%) and a two (*decision*: biased and unbiased) by three (*emotion*: angry, happy, neutral) by two (*block order*: first and second) repeated-measures ANOVA revealed that there were no differences in accuracy between decisions (F_1,15_ = 1.215, p = 0.288), emotions (F_2,30_ = 1.036, p = 0.367) or blocks (F_1,15_ = 0.394, p = 0.539), nor were there any interaction effects between decision and emotion (F_2,30_ = 0.968, p = 0.391), decision and block order (F_1,15_ = 1.384, p = 0.258), emotion and block order (F_2,30_ = 0.813, p = 0.453) (Fig. 1B).

To test whether emotions can differentially modulate the decisional strategy, we looked at the potential behavioral effects of a bias towards a particular auditory emotion (i.e. happy and angry voices, excluding neutral). This analysis did not take block order into account since the previous analysis did not reveal any effect. A three (*decision*: biased target, biased non-target, unbiased) by two (*emotion*: happy, angry) repeated-measures ANOVA on reaction times during correct trials revealed a main effect of decision (*F*_2,14_ = 4.05, *p* = 0.041) and a main effect of emotion (*F*_1,15_ = 55.46, *p* < 0.001; with angry voices being identified faster than happy voices), but no interaction effect (*F*_2,14_ = 0.24, *p* = 0.791). Pairwise comparisons (Bonferroni correction) revealed that *biased target* decisions were performed faster than *unbiased* decisions (*p* < 0.030), but there were no differences between *biased target* and *biased non-target* decisions (*p* = 0.278), or between *unbiased* and *biased non-target* decisions (*p* = 0.283).

### Functional brain data of the voice localizer scan

**Figure 2:**
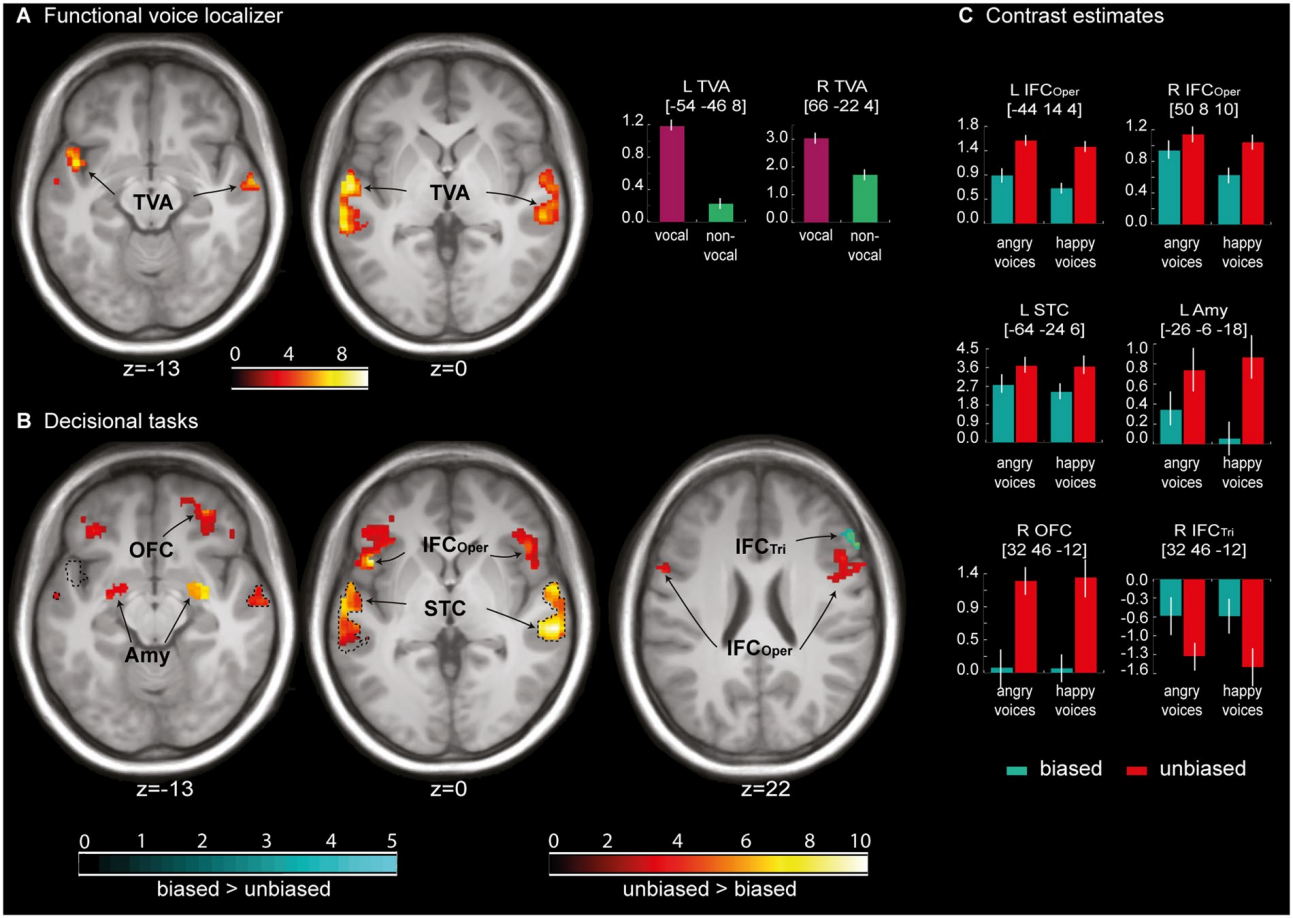
Functional activity for the localizer task and the different types of decision-making. (**A**) Whole-brain activations and contrast estimates for the functional voice localizer scan. (**B**) ROI brain activations for comparing biased and unbiased decisions on affective voices (i.e. excluding neutral voices). Unbiased decisions on affective voices (red to yellow gradient) activated the right OFC, bilateral STC, amygdala, and bilateral IFC_Oper_ extending into the IFC_Tri_, while biased decisions (blue to green gradient) activated the right IFC_Tri_. Dotted black lines mark the voice-sensitive areas as determined by the functional voice localizer scan. (**C**) Contrast estimates for peak activations in comparisons of biased and unbiased decisions on affective voices. The depicted color bars refer to T-maps. Abbreviations: IFC = inferior frontal cortex; TVA = temporal voice area; human = human speech and non-speech vocalizations; non-human = non-human vocalizations and sounds; IFC_Oper_, IFC_Tri_, IFC_Orb_ = pars opercularis, pars triangularis, and pars orbitalis of the IFC, respectively; OFC = orbito-frontal cortex; STC = superior temporal cortex; Amy = amygdala; Emo = emotional voices (angry and happy); Neu = neutral voices; L = left; R = right.

The voice localizer scan revealed bilateral activations in several regions in the bilateral STC when human speech and non-speech vocalizations were compared against non-human sounds and vocalizations (Fig. 2A).

### Functional brain data of the main experiment

**Figure 3:**
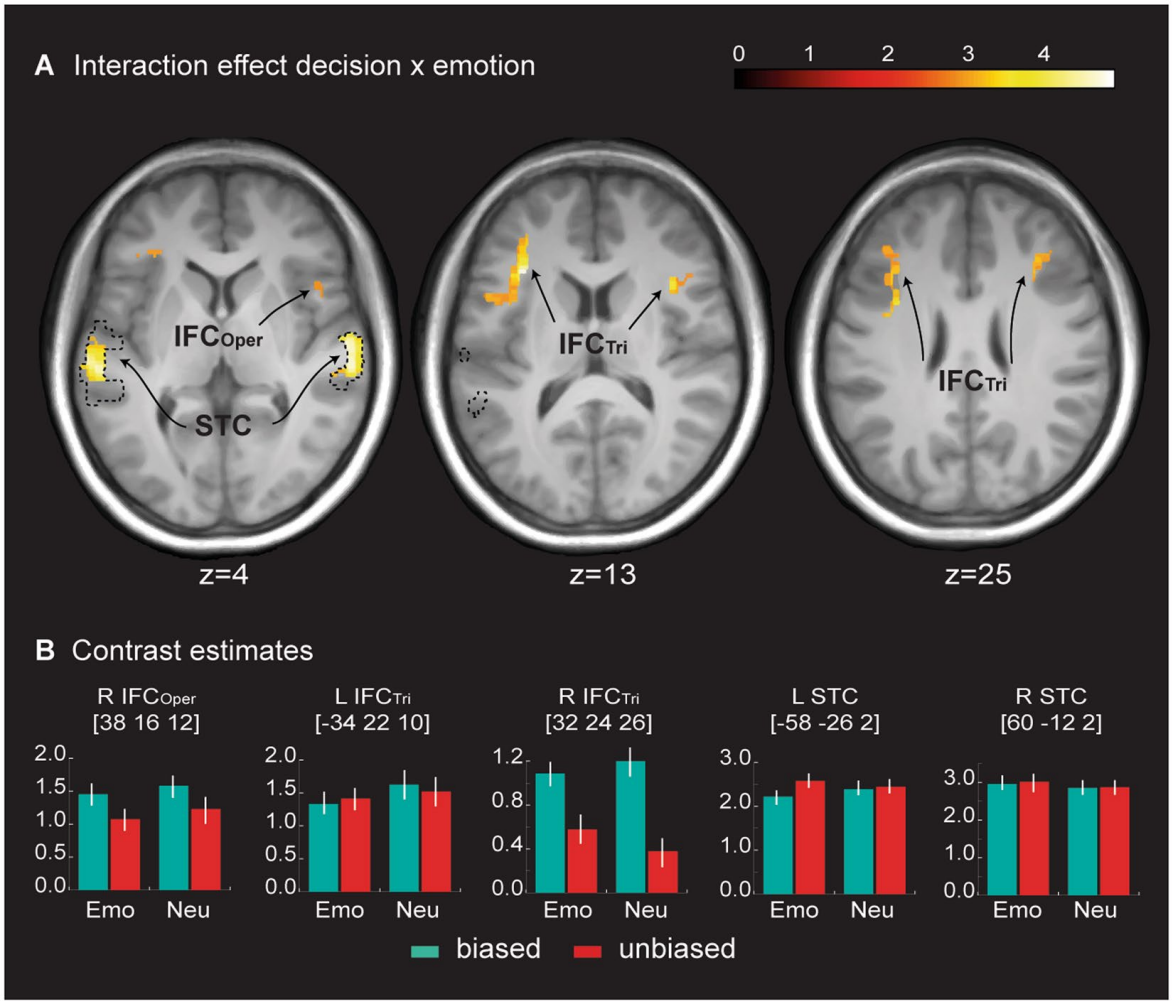
Functional activity in our regions of interest (ROI) analysis for the main effect of decision. The red to yellow gradient refers to unbiased versus biased decisions on *all* voices (angry, happy, and neutral). The blue gradient refers to biased versus unbiased decisions on *all* voices (angry, happy, and neutral). Dotted black lines mark the voice-sensitive areas as determined by the functional voice localizer scan. The depicted color bars refer to T-maps. For abbreviations, see Fig. 2.

Concerning the brain activations in our ROIs underlying the different types of decisions, we computed two different contrasts. First, because we wanted to explore the differences between types of decisions on affect alone, we compared the brain activity for affective voices (i.e. angry and happy voices, excluding neutral) between decisional tasks. This analysis was done because of the different status of the neutral voices during the biased and unbiased decision tasks. We found activations in the bilateral amygdala, bilateral STC, bilateral IFC_Oper_, and right OFC during unbiased decisions ([unbiased decision: affective voices > biased decision: affective voices]), whereas biased decisions on affective voices, compared with unbiased decisions ([biased decision: affective voices > unbiased decision: affective voices]), revealed activations in the right mid IFC_Tri_ (Fig. 2B,C, Table 1C,D). Second, for exploratory reasons, we also performed the same contrast, including all types of vocal affect (i.e. anger, happy, or neutral) perceived. Similar to the first analysis, unbiased versus biased decisions elicited activity with peak locations in the bilateral STC, amygdala, IFC_Oper_, and right OFC, while the reversed contrast between biased and unbiased decisions revealed distinct activations in the bilateral IFC_Tri_ (Fig. 3, Table 1A,B).

**Table 1:** Functional peak activations during the main experiment restricted to our ROIs.

**Region**	**Z-score**	**Cluster size**	**MNI coordinates**
**(A) Unbiased versus biased decisions on all voices (angry, happy, and neutral)**			
Left IFC_Oper_	7.82	973	−44, 14, 4
Right IFC_Oper_	6.47	911	58, 12, 14
Right IFC_Oper_	2.70	26	36, 8, 30
Right OFC	5.22	119	32, 46, −12
Left STC	Inf	776	−64, −24, 6
Right STC	Inf	550	60, −10, −6
Right Amy	6.61	116	28, −4, −20
Left Amy	5.26	57	−28, −4, −20
**(B) Biased versus unbiased decisions on all voices (angry, happy, and neutral)**			
Left IFC_Tri_	4.56	45	−38, 16, 24
Right IFC_Tri_	5.49	174	58, 24, 18
**(C) Unbiased versus biased decisions on affective voices (i.e. excluding neutral voices)**			
Left IFC_Oper_	7.454	1148	−44, 14, 4
Right IFC_Oper_	5.375	819	50, 8, 10
Right IFC_Tri_	2.997	20	40, 32, 28
Right IFC_Oper_	2.71	25	36, 8, 30
Right IFC_Tri_	2.33	13	32, 14, 26
Right OFC	4.75	134	32, 46, −12
Right STC	Inf	448	62, −28, 0
Left STC	Inf	353	−64, −24, 6
Left STC	3.56	16	−50, 10, −14
Left STC	3.52	12	−42, 30, −4
Right Amy	5.924	159	28, −4, −20
Left Amy	4.639	52	−26, −6, −18
**(D) Biased versus unbiased decisions on affective voices (i.e. excluding neutral voices)**			
Left IFC_Tri_	4.758	82	58, 24, 22
**(E) Interaction effect (decision × emotion interaction)**			
Left IFC_Tri_	4.39	361	−34, 22, 10
Right IFC_Oper_	3.49	40	38, 16, 12
Right IFC_Tri_	3.03	35	32, 24, 26
Right IFC_Oper_	3.09	16	24, 2, 28
Right STC	3.97	157	60, −12, 2
Left STC	3.83	281	−58, −26, 2

(A) Main effect of decision: unbiased versus biased decisions on all voices. (B) Main effect of decision: biased versus unbiased decisions on all voices. (C) Unbiased versus biased decisions on affective voices (i.e. excluding neutral voices). (D) Biased versus unbiased decisions on affective voices (i.e. excluding neutral voices). (E) Interaction between decision (unbiased versus biased) and emotion (affective voices versus neutral voices). For abbreviations, see Fig. 2.

**Figure 4:**
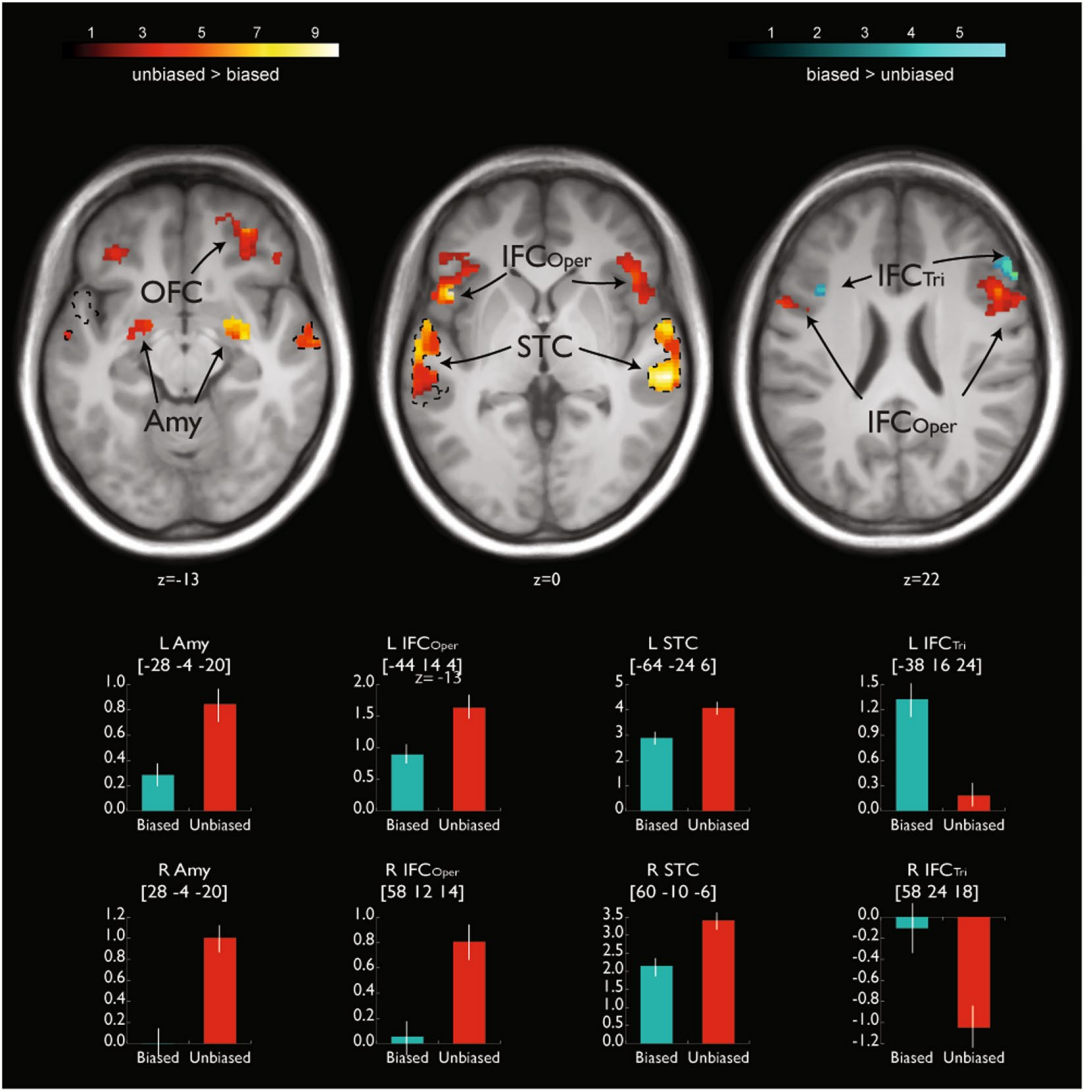
Functional activity for the processing of affective voices. (**A**) ROI activations pertaining to the interaction effect of decision and emotion ([unbiased decision: affective > neutral voices] > [biased decision: affective > neutral voices]) in the right IFC_Oper_, bilateral IFC_Tri_, and bilateral STC. Dotted black lines mark the voice-sensitive areas as determined by the functional voice localizer scan. (**B**) Contrast estimates for peak activations for the interaction between emotion and decision ([unbiased decision: affective > neutral voices] > [biased decision: affective > neutral voices]). The solid lines represent functional connections that overlap with connections found for unbiased decisions (see Fig. 4B). The depicted color bars refer to T-maps. For abbreviations, see Fig. 2.

Regarding the brain activity related to an emotion affect (i.e. processing of affective relative to neutral voices), we did not find significant activation in our ROIs across or within the decisional tasks. However, we found an interaction effect when we compared unbiased with biased decisions ([unbiased decision: affective > neutral voices] > [biased decision: affective > neutral voices]) in the bilateral IFC_Tri_ and right IFC_Oper_, as well as in the bilateral voice-sensitive areas in the STC (Fig. 4A,B, Table 1E).

### Functional connectivity

Peak activations found in our contrasts of interest served as seed regions in a psycho-physiological interaction (PPI) analysis to determine task-specific functional connectivity between regions. Specifically, we were interested in determining which of our ROIs, namely voice-sensitive areas in the STC, amygdala, OFC, and subregions of the IFC, showed significant increases in functional coupling with our seed regions during one type of decision making on affective voices compared to the other, as well as in relation to the emotional effect.

**Figure 5:**
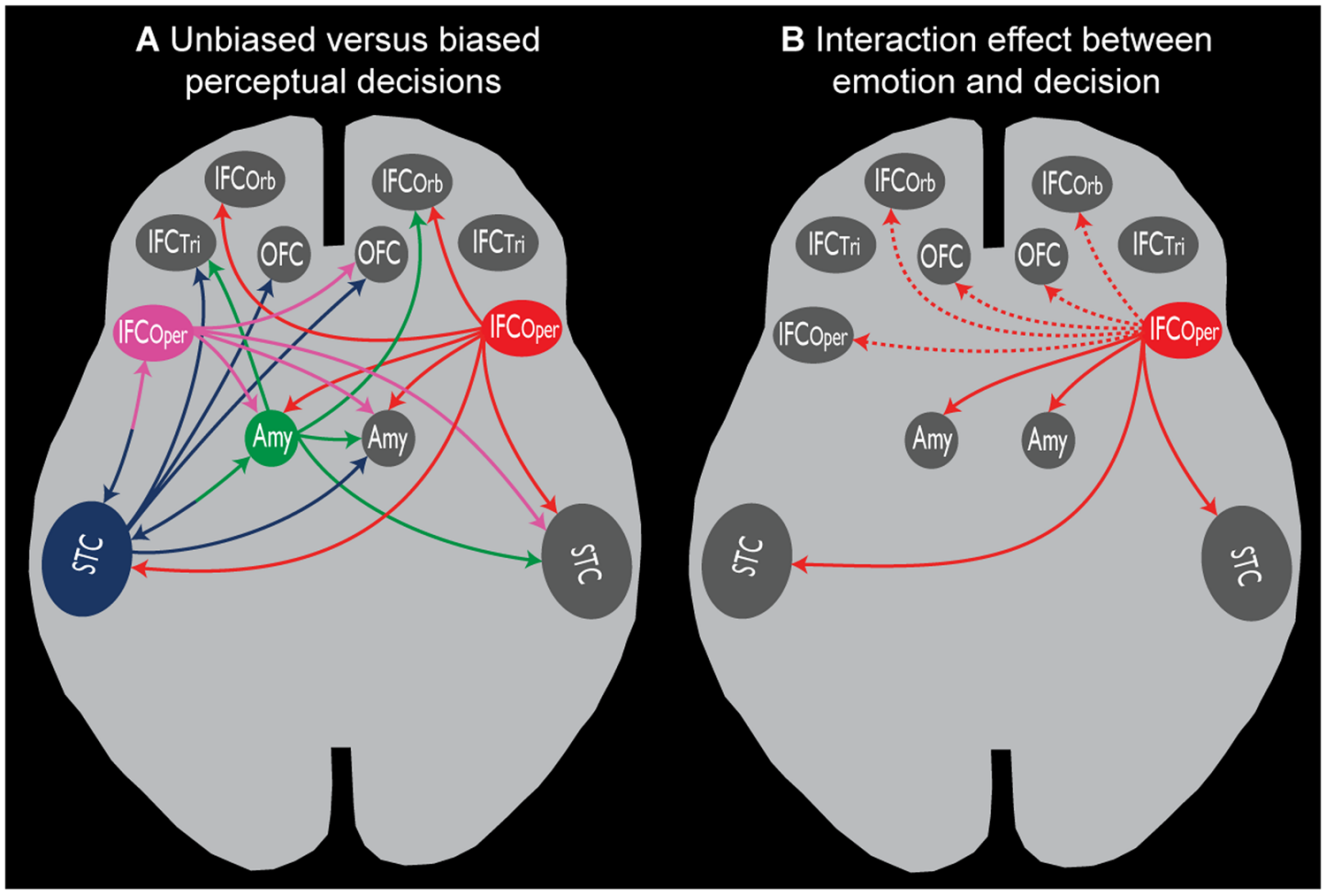
Functional connectivity during perceptual decision-making. (**A**) Task-induced functional connectivity during unbiased versus biased decisions on affective voices (i.e. excluding neutral voices). **(B)** Task-induced functional connectivity during processing of emotional versus neutral voices that is distinct for unbiased decisions. Seed regions and seed connectivity are shown in color. Target regions are shown in grey. The solid lines represent functional connections that overlap with connections found for unbiased decisions. For abbreviations, see Fig. 2.

**Figure 6:**
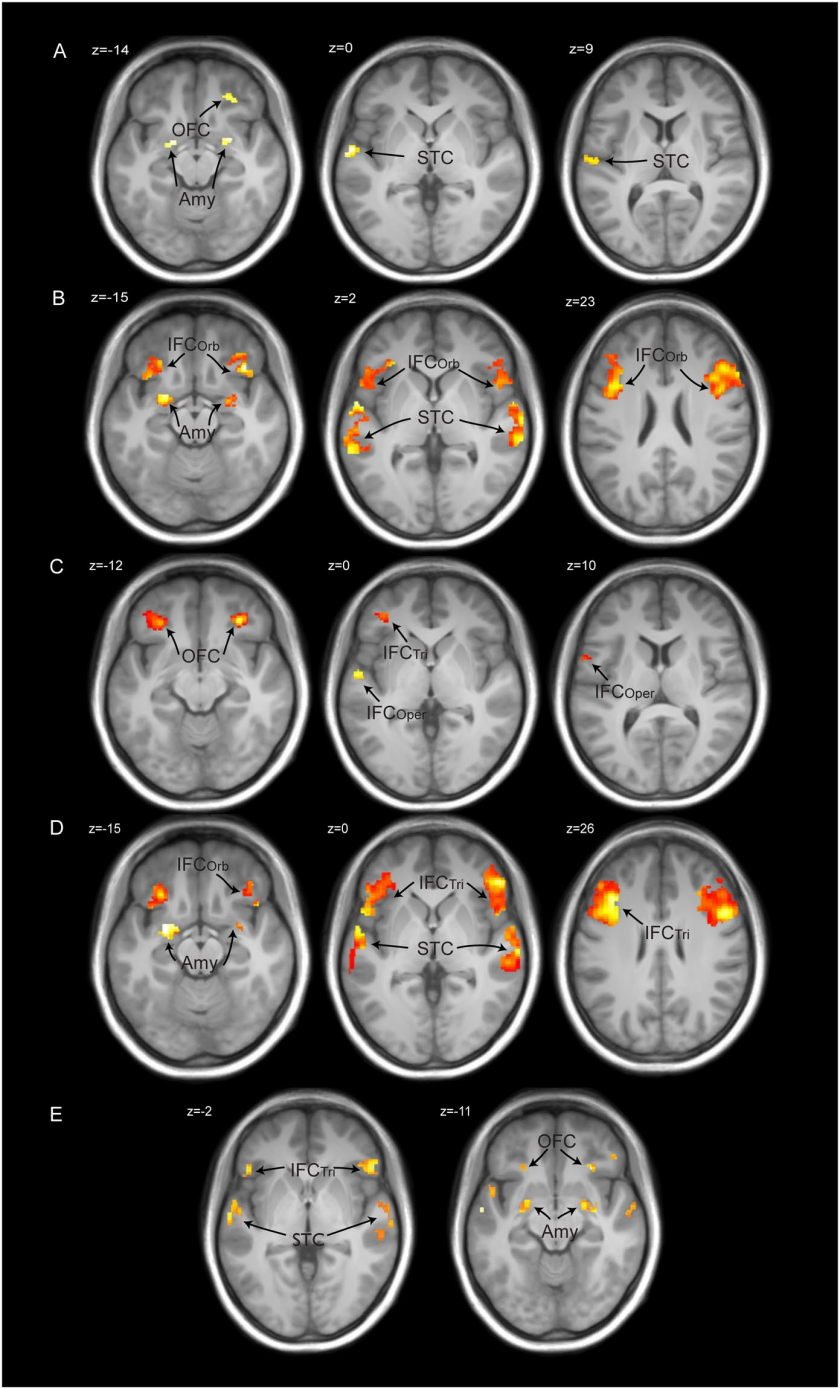
Task-induced functional connectivity related to the experimental factor decision. Functional connectivity during unbiased versus biased decisions on affective voices (i.e. excluding neutral voices) for seed regions in the (**A**) IFC_Oper_, (**B**) right IFC_Oper_, (**C**) left STC, and (**D**) left amygdala. (**E**) Task-induced connectivity related to the interaction between decision (unbiased versus biased) and emotion (affective voices versus neutral voices) with the seed in the right IFC_Oper_. For abbreviations, see Fig. 2.

During biased decision-making (excluding neutral voices), no other brain regions significantly coupled with our seed region in the right IFC_tri_ [MNI_xyz_ 58 24 22]. During unbiased decisions on affective voices, compared with biased decisions, bilateral IFC_Oper_ was jointly functionally coupled to the bilateral amygdala and the left STC (Figs 5,6A,B, Table 2A,B). Additionally, the right IFC_Oper_ [50 8 10] was distinctly connected to the bilateral IFC_Orb_ and the right STC, whereas the left IFC_Oper_ [−44 14 4] was distinctly connected to the right OFC. No regions were significantly coupled with the right OFC [32 46 −12], right amygdala [28 −4 −20], or right STC [62 −28 0]. However, peak activations in the left amygdala [−26 −6 −18] and the left STC [−64 −24 6] showed common functional coupling with the left IFC_Tri_ (Figs 5, 6C,D, Table 2C,D). Distinct connectivity was shown between the left STC and the left IFC_Oper_, left IFC_Tri_, and bilateral OFC. Additionally, the left amygdala was functionally connected to the right amygdala, right IFC_Orb_, right STC, and several regions across the left STC during unbiased decisions on affective voices compared to biased decisions.

**Table 2:** Task-induced functional connectivity related to the experimental factor decision restricted to our ROIs.

**ReRegion**	**Z-score**	**Cluster size**	**MNI coordinates**
**(A) Functional connectivity of left IFC**_**Oper**_			
Left STC	3.99	103	−58, −6, 0
Left Amy	3.25	32	−16, 0, −14
Right Amy	3.23	35	26, 2, −14
Right OFC	3.66	22	28, 34, −16
**(B) Functional connectivity of the right IFC**_**Oper**_			
Left STC	4.21	590	−56, −50, 12
Right STC	4.13	457	64, −18, 8
Left STC	3.35	49	−48, 22, −4
Left Amy	4.01	120	−24, 0, −18
Right Amy	3.29	92	30, −2, −12
Left IFC_Orb_	4.14	1391	−34, 18, −20
Right IFC_Orb_	4.03	1728	38, 24, −16
Left IFC_Orb_	2.69	33	−46, 42, −8
**(C) Functional connectivity of the left STC**			
Right OFC	5.45	120	30, 36, −14
Left OFC	3.75	142	−30, 34, −12
Left IFC_Tri_	3.45	51	−36, 40, 0
Left IFC_Oper_	2.98	31	−60, 4, 10
**(D) Functional connectivity of the left AMY**			
Right STC	4.58	513	60, −14, 4
Left STC	4.07	435	−56, −4, 2
Left STC	3.20	47	−50, 20, −2
Left STC	3.14	39	−60, −48, 10
Left Amy	3.61	134	−26, −2, −16
Right Amy	2.42	28	28, 0, −12
Right IFC_Orb_	4.31	2745	44, 18, −14
Left IFC_Tri_	4.03	2781	−38, 22, 28
**(E) Functional connectivity of the right IFC**_**Oper**_ **related to the decision × emotion interaction**			
Left IFC_Oper_	3.17	28	−32 4 30
Left IFC_Oper_	2.71	29	−40 20 32
Right IFC_Oper_	2.83	26	30 14 32
Left IFC_Tri_	3.42	118	−56 26 14
Left IFC_Tri_	3.37	100	−48 26 −2
Right IFC_Tri_	3.72	226	58 30 4
Right IFC_Tri_	2.87	60	44 22 28
Left OFC	3.08	33	−24 26 −10
Right OFC	3.63	35	28 26 −12
Left STC	135	3.94	−60 −8 −10
Left STC	131	3.03	58 −6 2
Left STC	48	3.00	−64 −24 10
Left STC	31	2.88	−54 −46 12
Right STC	26	2.87	56 −10 −12
Right STC	24	2.71	−52 10 −10
Left Amy	87	3.55	−22 0 −18
Right Amy	37	3.36	32 −8 −12

Functional connectivity during unbiased versus biased decisions on affective voices (i.e. excluding neutral voices) for seed regions in the (A) left IFC pars opercularis, (B) right IFC pars opercularis, (C) left STC, and (D) left amygdala. (E) Task-induced connectivity related to the interaction between decision (unbiased versus biased) and emotion (affective voices versus neutral voices) with the seed in the right IFC_Oper_. For abbreviations, see Fig. 2.

When making unbiased decisions on affective versus neutral voices, a pattern of functional connectivity emerged that was distinct from making biased decisions on affective versus neutral voices. Specifically, the right IFC_Oper_ [38 16 12] was functionally coupled with bilateral amygdala, STC, OFC, IFC_Tri_, and contralateral IFC_Oper_ (Figs 5, 6E, Table 2E).

## Discussion

In this study, we used fMRI to investigate the brain activation and functional connectivity in two forms of perceptual decision-making on affective voices: biased and unbiased decisions. We found extensive bilateral activation in the posterior IFC (IFC_Oper_) that was unique to unbiased decisions on affective voices. For biased decisions, the only region significantly more active was the right mid IFC (IFC_Tri_), located antero-lateral to the peak activation in the same region during unbiased decision making. This finding suggests a functional distinction within the IFC that reflects the differences in decisional strategies^36^, possibly linked to the diverse morphology and cytoarchitecture of the IFC^64^. Such functional differentiation in the IFC has two major implications. First, instead of a single brain region involved in decision formation that adjudicates what is being perceived, there could be distinct decision formation sub-regions within the IFC that respond to contextual demands. This roughly follows proposals of a rostral-to-caudal gradient in the frontal cortex, with more caudal regions being involved in abstract cognitive tasks^65^. Second, the functional distinction within the IFC could mirror the structural and functional pathways that target the IFC. Specifically, a dual-pathway model posits that a ventral stream from the STC to the more anterior IFC analyzes the global features of the voice while a dorsal stream from the STC to the more posterior IFC analyzes the dynamically varying features^37^. Global acoustic features, such as mean intensity and pitch, allow gross discrimination of the vocal emotion, on which biased decisions could be based^66^, whereas the unbiased decision could predominantly rely on varying finer-tuned features^23^. The need for biased and unbiased decisions to depend on different pathways and IFC sub-regions might come from how affective information is processed from voices. For instance, a one-step classifier of affective voices was found to be inferior to a two-step classifier consisting of a first stage for gross feature extraction, allowing the initial classification of the emotion in one of several broad categories, and a second stage for finer feature extraction and conclusive affective identification^67^. Perhaps global acoustic features are sufficient for the cursory processing of affective voices and their subsequent biased discrimination, whereas the finer level of unbiased decisions depends on both locally varying and global prosodic cues, thus necessitating stronger coupling between the different brain regions able to extract, process, and represent the affective values of auditory stimuli.

We additionally found activity in the right OFC as well as bilateral amygdala and STC for unbiased versus biased decisions. The amygdala plays an essential role in the accurate processing of the affective meaning of voices^52^, while the STC provides proper acoustic analysis of relevant voice features and their integration into an auditory percept^32^. The posterior IFC (IFC_Oper_) might then incorporate this additional affective and acoustic information on the way to a conclusive decision about the emotion that has been heard. Unbiased decisions might be further supported by the OFC, which seems to be involved in the late evaluative stages rather than the early sensory stages of emotional speech processing^68^.

Biased compared to unbiased decisions increased brain activity only in one region, namely the right IFC_Tri_. We note that this contrast revealed negative beta parameters for both types of decisions. In other words, there was less deactivation in the right IFC_tri_ during biased decisions compared to unbiased decisions. Such pattern of brain activity is not uncommon in neuroimaging^69^. However, the exact interpretation of this phenomenon is still debated, as it can equally reflect neural inhibition of task-irrelevant neural processing or hemodynamic compensatory mechanisms^70^. If we consider that the right mid IFC is involved in general behavioral and cognitive inhibition^43,71,72^, we can speculate that the IFC could also potentially contribute to unbiased decisions on affective voices by a markedly reduced inhibitory activity, in addition to the excitatory input to the amygdala and voice-sensitive areas in the STG that we see in our results.

The absence of significant differences in the BOLD activity of the STC and amygdala for the comparison between biased and unbiased decisions might suggest that biased decisions are largely accomplished without in-depth acoustic and emotional analysis of the voices. If we conceive of the general decision-making process as a “random walk” in which sensory information in favor of one alternative or another is gradually accumulated toward a decision threshold^73,74^, then we can consider two explanations for our imaging and behavioral results. First, the anticipation of the favored perceptual alternative (i.e. the target emotional voice) during biased decisions might set the origin of the random walk closer towards the threshold for biased choice options^12,30,75^. Under this account, less bottom-up sensory information needs to be accumulated for accurate perception and for the decision threshold to be reached. Conversely, the diffusion processing for unbiased decisions begins equidistant from the decisional thresholds for the three choice options, thus requiring on average more information to be accumulated to confirm a percept, which then triggers extensive processing in auditory and limbic regions. Second, it is possible that the rate of the acquisition of sensory information is actively modulated by prior expectations^30,76^. Specifically, the decisional bias increases the salience of the target stimuli, leading to their disproportionally faster processing in the first stages of evidence accumulation, and significantly slower processing in the later stages. Such non-linear mechanism of information accumulation has been proven in sequentially presented evidence of highly salient stimuli^77–79^, similar to the biased emotional voices in our study. De Lange *et al*.^77^ found that the brain activity in regions of evidence accumulation was inversely related to the amount of accumulated evidence, such that when more evidence had been accumulated during initial stages of perception, neural activity was attenuated for later stages of perception. It is possible that the restricted pattern of neural activity and connectivity in our results during biased decisions comes also from the limited temporal resolution of the fMRI that can only capture the linear mechanism of evidence accumulation during unbiased decisions. Future studies using more sensitive techniques such as magnetoencephalography^77^ can provide the much needed temporal resolution for the investigation of biased and unbiased decisions.

Together, the data above highlight the notion that unbiased decisions rely on a distributed neural network that provides relevant information for a proper decision, while biased decisions mostly rely on functional mechanisms provided by a single frontal region. In terms of functional brain connectivity, we did not find significant increased coupling between our ROIs during biased compared with unbiased decisions on affective voices. Instead, we found that the IFC, amygdala, and STC were significantly coupled during unbiased decisions on affective voices compared to biased decisions, thus pointing to an extended neural network rather than a single region underlying these decisions. This connectivity network revealed three notable features. First, bilateral IFC_Oper_ was primarily a source of connectivity towards our ROIs, which might then drive co-activation in other regions. Second, the left amygdala and STC were both targets and sources of functional connectivity, while the right amygdala and STC were solely target regions. Third, there appears to be a functional connectivity loop in the left hemisphere between the IFC_Oper_, STC, and amygdala, and this loop also involves the OFC. Concerning the functional meaning of this network, we propose that the IFC detects task difficulty^17^ and consequently initiates or enhances acoustic and emotional processing in the STC and amygdala, respectively, via top-down influences^80^. Moreover, this enhanced processing is accompanied by a series of functional cross-talks between the IFC, STC and the amygdala. The STC might interact with the left amygdala in order to incorporate affective information into an integrated percept of the affective voice^81^, and this emotional gestalt is made available to higher order frontal regions, such as the IFC^82^ and the OFC^83^, which support the proper evaluation of the affective meaning of voices.

We additionally found activity in bilateral IFC and STC in the interaction between decision and emotion only for unbiased versus biased decisions. Therefore, the affective decoding of voices in the STC and IFC^36,84^ becomes relevant only when one is confronted with more complex cognitive mechanisms of decisions that require proper affective classification when given several choice options. The absence of an emotion effect during biased decision-making suggests that the success of this type of decision depends on less acoustic and emotional information compared to the more complex unbiased decisions, in line with the drift-diffusion model^61^. For example, happy voices could be discriminated from other affective voices by taking into account only simple acoustic features such as fast onsets and tone attacks of the vocalization^85^ or spectral variability^86^ without having to analyze the acoustical features related to emotion at a more fine-grained level.

Interestingly, the original contrast for the interaction between emotion and decision did not reveal activity in the amygdala, but the functional connectivity analysis revealed significant coupling of the amygdala with the posterior IFC as the source. This indicates that the affective decoding in the amygdala does not occur instantaneously when confronted with an auditory emotion but rather seems to be triggered by the IFC when required by the complexity of the decisional task^80^. The affective decoding during more complex decisions therefore depends on a widespread functional network that provides emotional (amygdala) and acoustic information (STC), as well as evaluative processing (IFC and OFC). This pattern of connectivity for the interaction between decision and affect appears to partly overlap with the connectivity pattern between decisions on affective voices. The overlap specifically concerns the right IFC_Oper_ as a source of connectivity to the bilateral amygdala and bilateral STC, indicating that during unbiased decisions, these connections initiate the processing of the affective meaning of voices, furthering functional loops in the left hemisphere as outlined earlier.

### Strengths and limitations

Because unbiased and biased decisions point to different strategies commonly used to identify stimuli in the environment – in our case, different emotional voices, we wanted to keep their characteristics intact and not artificially control for task differences more than we needed to. We operationalized these decisional strategies as “A or B or C” and “A or not A (i.e. B or C)”, respectively. We called *unbiased decisions* as such in order to contrast them against *biased decisions*. A prototypical unbiased decision would involve the free choice of answers in the absence of any predefined categories. Such prototypical decisions exist in behavioral research^87^ but in the present study the paradigm has been adapted to the fMRI environment where only a button box with a limited number of choices could be used, whereas a verbal output would likely have introduced motion artefacts. Research has shown comparable levels of accuracy between free-choice and fixed-choice identification of emotions^87,88^.

While it might seem that the two decisions fundamentally differ in the number of choice categories (i.e. three versus two), we argue that the biased decision is not a traditional two-alternative forced-choice task despite the presence of only two possible button presses^59^. Participants do not perceive two types of stimuli as the “not A” category is not a stimulus class in itself but rather an ad hoc construct. To successfully perform the task, the participants must first identify the stimulus as pertaining to its original class (i.e. angry, happy, neutral voices) and subsequently allocate it to “target” or “non-target”.

One can argue that biased and unbiased decisions differ on too many dimensions and therefore involve different levels of complexity. If that were true, then we would expect to see behavioral and neural correlates of these differences. Task complexity can refer to a multitude of things such as cognitive mechanisms, task difficulty or motor output. Although reaction times were slower for biased compared to unbiased decisions, accuracy rates would be a better indicator for task difficulty and the two decisions did not differ in this regard. This suggests that the two tasks diverge not in difficulty but rather in the underlying computational mechanisms or motor preparation times. Moreover, participants signaled neutral voices with a double button press during unbiased trials and with a single button press during biased trials. Although we mainly focused our analyses on emotional trials only (i.e. angry and happy voices), the different motor scheme during neutral trials could have induced effects pertaining to motor preparation times that spilled over the rest of the trials. Alternatively, it can be argued that the requirement of simultaneously pressing two buttons artificially increased the salience of unbiased trials compared to biased trials. However, when comparing biased and unbiased decisions on neutral voices trials (i.e. single versus double button presses), we did not find significant differences in the neural correlates. Furthermore, we found no main effect of this motor scheme on reaction times (F_1,15_ = 0.329, p = 0.574) or accuracy (F_1,15_ = 0.303, p = 0.590) during neutral trials. In light of this, it appears that we have a double dissociation: biased and unbiased decisions showed no differences in the neural correlates nor in reaction times during neutral trials where the motor output diverges the most, but they show significant differences during emotion trials where the motor output is identical. Therefore, the different motor output of the two tasks did not significantly alter our findings. Together with the similar accuracy rates that indicate comparable levels of difficulty, we propose that biased and unbiased decisions should differ in the underlying computational mechanisms. Specifically, biased decisions rely on a gross extraction of features, which allows for the quick identification of the voice, a process that is superficial and yet sufficient for the subsequent classification of the stimulus into a broader category, i.e. target or non-target. Conversely, unbiased decisions require a more extended functional network, which provides in-depth acoustic and emotional information.

During biased and unbiased trials, participants listened to identical stimuli, the varying element being the decisional strategy that is enforced through instructions. In this regard, we believe that the number of choice categories are paramount to ensure the conceptual distinction between the tasks and, therefore, should not be controlled for. Summerfield and Koechlin^56^ introduced the same decisional bias as we do but in a non-emotional visual task while controlling for the number of buttons. Specifically, their participants had to decide between two Gabor patches using an “A or not-A” decisional strategy (i.e. same as ours) or using an “A or B” strategy. While we appreciate their desire to control for the number of buttons across decisions, we also strongly believe that such paradigm cannot account for the individual strategies that each participant uses. As such, there is no way of determining whether the participant truly used the designated decisional strategy within a particular trial or whether they interchangeably used an “A or not A” or an “A or B” across trials. Having the same number of choices makes it possible for the participant to use the opposite strategy, either intentionally (e.g. personal preference to ensure speed or accuracy) or unintentionally (e.g. a carryover effect introduced by the randomization process). In fact, the authors^56^ report virtually identical reaction times across “A not A” and “A or B” decisions. Contrary to their approach, we introduce a third choice-category in order to make it more difficult for the participant to use a different strategy other than the designated one and, as discussed above, such manipulation did not introduce unwanted behavioral and neural results.

In conclusion, our study shows that the presence or absence of priming during perceptual decision-making on vocal emotions induces different patterns of functional activity and connectivity in the IFC and OFC. This has direct implications for the research into perceptual decision-making that so far has mainly focused on two-alternative forced-choice tasks^3^, ignoring the ecological variety of perceptual choices that we make on a constant basis. Different circumstances require different perceptual choices and our results show that the brain adapts to these contextual demands. Scientific inquiry should therefore acknowledge the diversity of perceptual decision-making when interpreting and extrapolating results from specific paradigms. Because of the nature of our stimuli and experimental design, we cannot make any claims as to whether the pattern of functional activity and connectivity is unique to the auditory processing of emotions. Future studies should incorporate different types of modalities of varying degrees of perceptual complexity.

## Methods

In the present study, we introduced two types of perceptual decision-making. Biased and unbiased decisions are two strategies with high ecological validity used to identify stimuli in the environment – in our case, vocal emotions. We note that the term *bias* is used in two ways in the computational neuroscience literature. First, from the viewpoint of sensory information, bias refers to the role that context and prior information have on perception and perceptual choices. Specifically, perception is seen as an inferential process, such that incoming sensory information is not analyzed de novo but interpreted based on prior information^24^. Second, from the viewpoint of the decisional strategy, the terms biased and unbiased decisions to refer to prior expectations regarding some of the choice categories^61,89^. During biased decisions, in contrast to unbiased, the participant is asked to focus on a specific emotion category, discriminating each incoming stimulus into either target or non-target. The current study aimed at investigating biased decisions on vocal emotions from the second perspective as this is a prevalent ecological perspective in decision-making on emotions. Furthermore, we call *unbiased decisions* as such in order to contrast them against *biased decisions*. A prototypical unbiased decision would involve the free choice of answers in the absence of any predefined categories. Such a paradigm exists in behavioral research but in this study, it has been adapted to the fMRI environment.

### Participants

Sixteen healthy volunteers (five males; mean age 24.81 years, SD = 4.41, age range 19–34) took part in the experiment. The participants had normal hearing abilities and normal or corrected-to-normal vision. No participant presented a neurological or psychiatric history. All participants gave informed and written consent for their participation. The local ethics committee of the University of Geneva approved all experimental protocols and methods of data collection, data handling and analysis. All methods and experimental protocols were performed in accordance with the guidelines and regulations of the local ethics committee of the University of Geneva.

### Stimuli

The stimulus material consisted of speech-like but semantically meaningless two-syllable words from the Geneva Multimodal Emotion Portrayal (GEMEP) database^90,91^. These pseudowords were 16-bit recordings sampled at a 44.1 kHz sampling rate and with a mean duration of 675 ms (SD = 192ms). During the experiment, stimuli were presented at 70 dB SPL. One male and one female speaker spoke six different pseudo-words (“belam”, “lagod”, “namil”, “nodag”, “nolan” and “minad”) in an angry, happy, or neutral tone. These twelve pseudo-words (six per gender) were selected from a behavioral evaluation of sixteen pseudo-words (eight per gender) out of the GEMEP database on six emotion scales (arousal, sadness, joy, anger, fear, neutral) performed by 12 participants (9 females, mean age 27.17 years, SD 4.39 years). We selected those pseudo-words that were most consistently evaluated as angry (*F*_1.30,14.25_ = 481.70, *p* < 0.001, Greenhouse-Geisser (GG) corrected), happy (*F*_1.43,15.76_ = 146.46, *p* < 0.001, GG corrected), and neutral (*F*_1.33,14.61_ = 447.29, *p* < 0.001, GG corrected), respectively. One-way repeated-measures ANOVA revealed that there was a statistically significant difference in perceived arousal scores among the angry, happy, and neutral voices (*F*_2,22_ = 202.91, *p* < 0.001). Pairwise comparisons revealed that arousal scores for angry and happy tones were significantly higher than scores received by neutral tones (*p* < 0.001), but they did not differ from each other (*p* = 0.351).

To localize human voice-sensitive regions in the bilateral STC, we presented in a separate run (i.e. referred to as a voice localizer scan) 500 ms sound clips consisting of 70 human speech and non-speech vocalizations, and 70 non-human vocalizations and sounds (animal vocalizations, artificial sounds, natural sounds) presented at 70 dB SPL^11^.

### Experimental design

For the fMRI experiment, we used an event-related design with two runs of unbiased decisions (i.e. three-alternative forced-choice decision of whether the voice was angry, happy, or neutral) and two runs of biased decisions (i.e. decision about the presence or absence of a target emotion). For example, in the two biased decision runs, the same participants received a “happy or not happy” set of instructions and an “angry or not angry” set instructions, respectively. For unbiased decisions, the participants received a “happy or angry” and an “angry or happy” set instructions, respectively. In addition, we counterbalanced these four runs using a Latin Square method, such that each type of instruction was always followed by another type an equal number of times. Within each run, 36 voices (12 per emotional tone) were presented twice inside distinct trials in a partially randomized order, so that the same type of emotion was never presented more than three times in a sequence. Each trial started with a visual fixation cross (1×1°) presented on a black background, followed by the presentation of a voice and then by a jittered blank screen until the onset of the next trial. The jitter for the fixation cross was randomly chosen an equal amount of times from an array of six possible durations (800 ms, 840 ms, 880 ms, 920 ms, 960 ms and 1000 ms). The jitter for the blank screen following the stimulus was randomly chosen an equal number of times from an array of six possible durations (3000 ms, 3400 ms, 3800 ms, 4200 ms, 4600 ms and 5000 ms). The fixation cross prompted the participant’s attention and remained on the screen for the duration of the auditory stimulus. The total length of each run was approximately seven minutes. The stimuli were presented binaurally through MRI-compatible headphones. The participants listened to each voice and made a corresponding button press as soon as they could identify the emotional tone (Fig. 1A). During unbiased decision blocks, participants used both index fingers to identify the emotion heard, as follows: one button press for happy voices, one button press for angry voices, and simultaneous button presses for neutral voices. During biased decision trials, participants pressed one button for the target emotional tone (either happy or angry) and another for the non-target emotional tone (neutral was always a non-target sound). Task blocks alternated across the experiment, and block order and response buttons were counterbalanced across participants.

For the voice localizer, each sound clip was preceded by a 500 ms fixation cross and followed by a jittered 3550–5000 ms (from a uniform distribution in 50 ms steps) gap before the onset of the next stimulus. To maintain participants’ attention, 10 randomly chosen sounds were repeated twice in a row, and participants had to detect and indicate each repetition by an index finger button press.

### Image acquisition

We recorded functional imaging data on a 3-T SIEMENS Trio System (Siemens, Erlangen, Germany) using a T2*-weighted gradient multiplexed echo-planar imaging (M-EPI) pulse sequence^92^ with an acceleration factor of four, 3 mm isotropic resolution, 36 slices in a 64×64 matrix, 20% distance factor, TR/TE = 650/30 ms, and FA = 50°. Structural images had 1 mm isotropic resolution (192 contiguous 1 mm slices, TR/TE/TI = 1900/2.27/900 ms, FoV = 296 mm, in-plane resolution of 1×1 mm). For the voice localizer scan, we used a partial volume acquisition of 28 slices (TR/TE = 650/30ms, thickness/gap = 2/0.4 mm, FoV = 192 mm, in-plane resolution 2×2 mm, FA = 54°) aligned obliquely to the AC-PC plane to cover all portions of the STC from anterior to posterior. Additional physiological data (heart rate and respiration) were recorded with the MP150 Biopac Systems acquisition system (Santa Barbara, CA).

### Data analysis

Preprocessing and statistical analyses of functional images were performed with Statistical Parametric Mapping software (SPM8, Welcome Department of Cognitive Neurology, London; www. fil.ion.ucl.ac.uk/spm). Functional data were first manually realigned to the AC-PC axis, followed by motion correction of the functional images. Each participant’s structural image was coregistered to the mean functional image, and then segmented to allow estimation of normalization parameters with the DARTEL algorithm^93^. Using the resulting flow fields, the anatomical and functional images were spatially normalized to the Montreal Neurological Institute (MNI) stereotactic space. The functional images for the main experiment were resampled into 2 mm^3^ voxels. All functional images were spatially smoothed with a 6 mm full-width half-maximum (FWHM) isotropic Gaussian kernel.

For the first-level analysis of the voice localizer, all trials were modeled with a stick function aligned to the onset of each stimulus, which was then convolved with a standard hemodynamic response function (HRF). Simple contrasts for vocal and non-vocal stimuli were then taken to a random-effects group-level analysis to determine voice-sensitive regions along the STC in both hemispheres.

For the first-level statistical analyses of our main experiment, we similarly used a general linear model in which each trial was modeled with a stick function set at the onset of the auditory stimuli and in which trial-by-trial reaction times were used as a parametric regressor (see Supplementary Fig. S1). By default, SPM8 performs orthogonalization according to the previous regressor. The stick functions were then convolved with a canonical HRF. Because neutral voices were always non-target in discrimination trials, we treated them as a single condition, regardless of whether the target emotion was angry or happy prosody. This yielded eight experimental conditions (see Supplementary Fig. S2), three for unbiased decisions (*angry*, *happy*, *or neutral voices*) and five for biased decisions, as follows: decisions on angry voices when the bias was towards angry voices (i.e. *angry target*) or towards happy voices (i.e. *angry non-target*), decisions on happy voices when the bias was towards happy voices (i.e. *happy target*) or towards angry voices (i.e. *happy non-target*), and discrimination of neutral voices (i.e. *neutral non-target*). For each of the eight experimental conditions, we created a regressor for the correct trials of each type of emotion and one additional regressor for all the incorrect trials collapsed across emotions. Accuracy of the classification of neutral voices was defined as synchronous button presses, regardless of which button was pressed first. Consequently, if only one button was pressed, or no presses were made, the respective neutral trials were labeled as incorrect. Reaction times for classification of neutral voices were calculated based on which of the two buttons were pressed first. Additionally, six motion correction parameters and four physiological parameters were included as regressors of no interest to account for signal changes not related to the conditions of interest. Physiological parameters were analyzed by using Retrospective Image Correction^94^, as implemented for MATLAB (http://cbi.nyu.edu/software). Simple contrasts for each experimental condition and for each participant were then taken to a random-effects group analysis by using a flexible factorial ANOVA, following the guidelines of Jan Gläscher and Darren Gitelman^95^. Specifically, for «Factors», we used subject as Factor 1 (Independence set to Yes and Variance set to Equal) and condition as Factor 2 (Independence set to No and Variance set to Equal). For «Subjects», we provided all images for each subject based on a matrix that maps a subject’s image onto the different factor levels. Under «Main effects & Interactions», we included a main effect for Factor 1 (16 levels) and a main effect for Factor 2 (8 levels), and no interactions. In the resulting second-level design matrix (see Supplementary Fig. S2), we looked at the zero-sum linear combination of the condition parameters of interest. All group results were thresholded at a combined voxel threshold of *p* < 0.05 (FDR corrected) and a cluster extent threshold of *k* = 10.

Our main focus was to compare biased with unbiased decisions on emotions. As a result, our design was unbalanced, with neutral voices only being non-target during biased discrimination blocks. Such an unbalanced design resulted from needing neutral voices as a third category in order to make the conceptual distinction between biased and unbiased decisions, while aiming to keep the scanning time to a minimum. Consequently, we first explored the main effects of task by comparing functional activations during unbiased decisions on all voices relative to biased decisions, irrespective of the type of emotion. In a second analysis, we contrasted the two types of decisions on affective voices directly (i.e. excluding neutral voices). Additionally, we contrasted affective voices with neutral voices within and across decisional tasks, and we examined the interaction between affective voices and the type of decision in order to reveal emotion effects that are specific for each type of decision making.

Because of our a priori interest in the IFC, the OFC, STC, and amygdala, we restricted our analyses to these predefined regions of interest (ROIs) by applying masks on whole-brain results. We opted for this approach because it is more conservative than small-volume correction. We used the Automated Anatomical Labeling (AAL) atlas^96^ to define ROIs for the bilateral IFC, OFC, and amygdala. The three bilateral subdivisions of the IFC, namely pars opercularis (IFC_Oper_), pars triangularis (IFC_Tri_), and pars orbitalis (IFC_Orb_), were combined with the bilateral OFC into a single mask of bilateral IFC-OFC. For the bilateral STC, we created functional ROIs of voice-sensitive auditory cortex identified from the voice localizer scan.

To reveal functional connectivity between our ROIs, we performed a psycho-physiological interaction (PPI) analysis^97^. For each of our group-level contrasts of interest, peak activations found in bilateral IFC, OFC, STC, and amygdala were chosen as seed regions, from which we extracted the first eigenvariate in a 3 mm radius sphere at the level of each participant. For each seed region, the PPI analysis was set up as a general linear model that included three regressors. The first regressor contained the extracted and deconvolved time course of functional activation within a seed region in each of the four functional runs (the physiological variable). The second regressor included the comparison between unbiased and biased decisions on affective voices (the psychological variable). In other words, we created a time course regressor for the task, including as many sampling points as for the physiological variable. The values in the second regressor were set to “1” for trials of biased decisions on angry and happy voices (excluding neutral) and “−1” for trials of unbiased decisions on angry and happy voices (excluding neutral). Only trials were included in the PPI analysis where participants gave a correct response. A separate analysis included a regressor for trials of unbiased decisions set to “1” and trials of biased decisions set to “−1” (please see Supplementary Fig. S3). Finally, the third regressor included the interaction between the first two regressors, which was created by a point-by-point multiplication of the time course for the physiological variable and the time course for the psychological variable. This interaction regressor was the only regressor of interest, whereas the physiological variable and the psychological variable served as co-variates of no interest in each PPI analysis. Individual results for these PPI analyses for each group seed region were entered into separate second-level flexible factorial analyses, where target regions of functional connectivity were limited to our ROIs. All resulting statistical maps were thresholded at a combined voxel threshold of *p* < 0.05 (FDR corrected) and a cluster extent threshold of *k* = 10.

### Data availability

Under the Swiss guidelines of data protection (Ordinance HFV Art. 5), the datasets generated and analyzed during the current study can be made available from the corresponding author on a case by case basis.

## Electronic supplementary material

Supplementary Material
