# Supplementary material for: Biased and unbiased perceptual decision-making on vocal emotions
Source: Sci Rep. 2017 Nov 24;7:16274. doi: 10.1038/s41598-017-16594-w (PMC5701116; doi:10.1038/s41598-017-16594-w)
Supplement: Supplementary file 1 — Supplementary Material [file 41598_2017_16594_MOESM1_ESM.pdf]

Scientific Reports

Supplementary Information

Biased and unbiased perceptual decision-making on vocal emotions.

Mihai Dricu, Leonardo Ceravolo, Didier Grandjean, and Sascha Frühholz

---

Figure S1. **First-level design matrix for a typical participant**. The four runs are analyzed together. **Run 1** = unbiased decisions on “angry or happy” voices, **Run 2** = unbiased decisions on “happy or angry” voices. **Run 3** = biased decisions on “angry or not angry” voices. **Run 4** = biased decisions on “happy or angry” voices. Each run consists of eighteen columns in the following order: correct decisions on angry voices, their parametric regressor (trial-by-trial reaction times), correct decisions on happy voices, their parametric regressor (trial-by-trial reaction times), correct decisions on neutral voices, their parametric regressor (trial-by-trial reaction times), all incorrect decisions during that run), their parametric regressor with reaction time and, finally, ten regressors of no interest (six motion correction parameters and four physiological parameters).

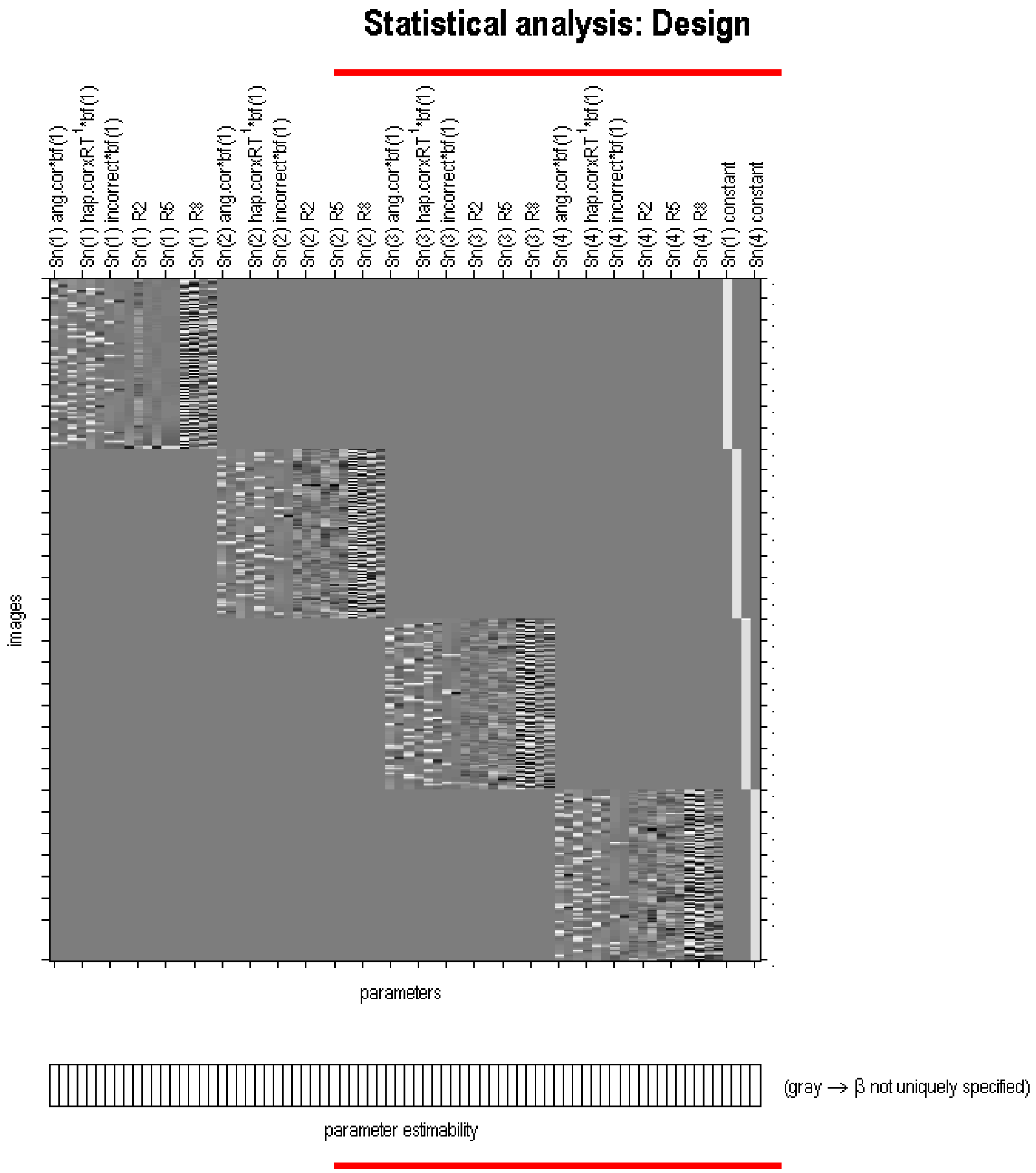

Design description...

Basis functions : hrf
Number of sessions : 4
Trials per session : 4 4 4 4
Interscan interval : 0.65 {s}
High pass Filter : Cutoff: 128 {s}
Global calculation : mean voxel value
Grand mean scaling : session specific
Global normalisation : Scaling

Figure S1

Figure S2. **Second-level flexible-factorial design matrix for 16 participants**. Conditions 1-3 refer to unbiased decisions while conditions 4-8 refer to biased decisions: ***condition 1*** = unbiased decisions on angry voices, ***condition 2*** = unbiased decisions on happy voices, ***condition 3*** = unbiased decisions on neutral voices, ***condition 4*** = biased decisions on angry voices when target voice was angry (i.e. angry target), ***condition 5*** = biased decisions on happy voices when target voice was angry (i.e. happy non-target), ***condition 6*** = biased decisions on angry voices when target voice was happy (i.e. angry non-target), ***condition 7*** = biased decisions on happy voices when the target voice was happy (i.e. happy target), ***condition 8*** = biased decisions on neutral voices, regardless of target voice. In the flexible factorial design, subjects and conditions were included as separate regressors (Factor 1 and Factor 2, respectively).

Statistical analysis: Design

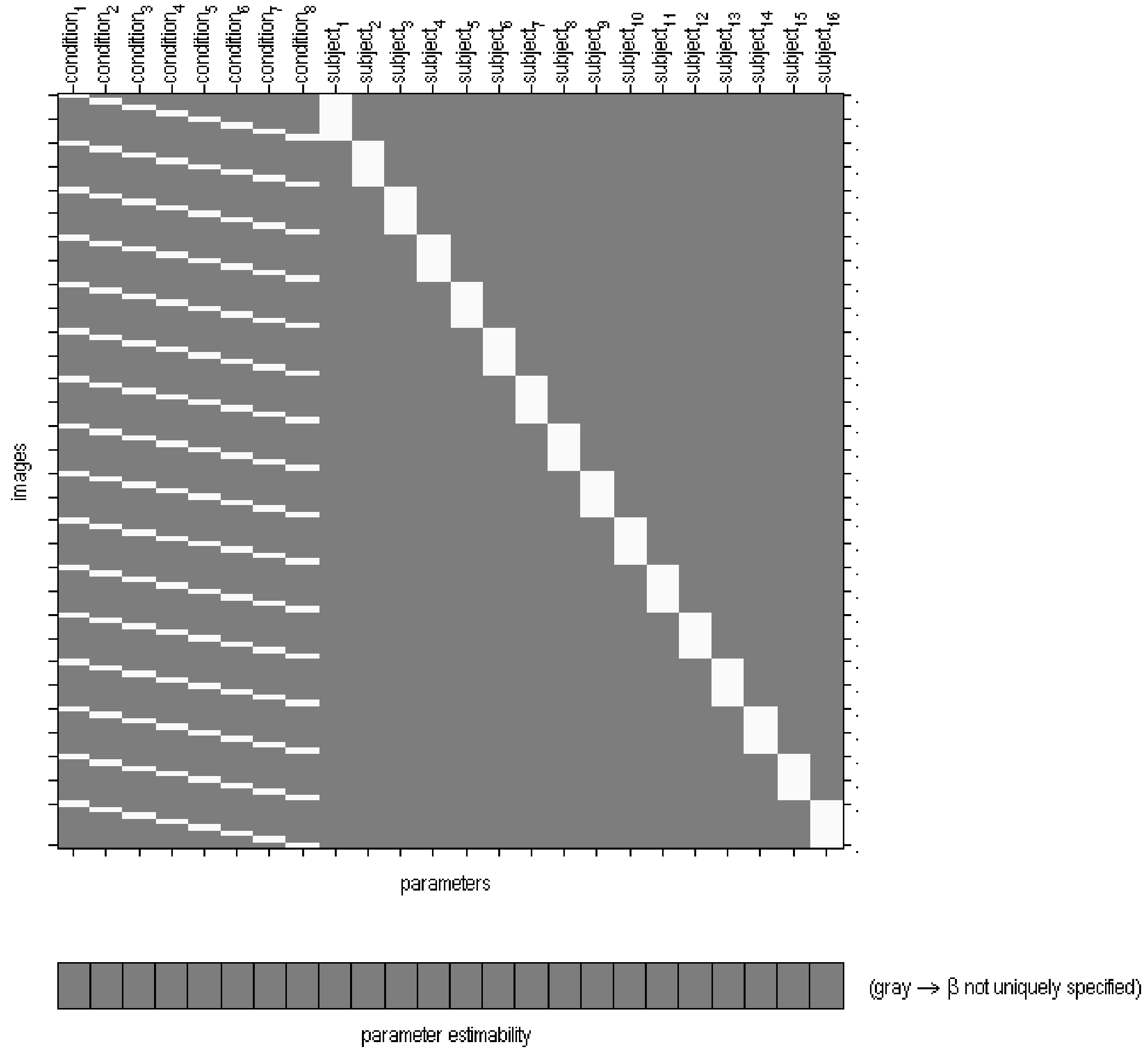

Design description...

Design : Flexible factorial
Global calculation : omit
Grand mean scaling : <no grand Mean scaling>
Global normalisation : <no global normalisation>
Parameters : 8 condition, +0 covariate, +16 block, +0 nuisance
24 total, having 23 degrees of freedom
leaving 105 degrees of freedom from 128 images

Figure S2

Figure S3. Example of the design matrix for the PPI analysis with the left IFC_{Oper} as a seed region. The four conditions refer to the four runs of collected data: **condition 1** = unbiased decisions on angry or happy voices; **condition 2** = unbiased decisions on happy or angry voices; **condition 3** = biased decisions on angry or not angry voices; **condition 4** = biased decisions on happy or not happy voices. In this particular example, for the unbiased > biased contrast (psychological variable in the PPI model) the first two conditions had “1”, while the last two conditions had “-1”.

Statistical analysis: Design

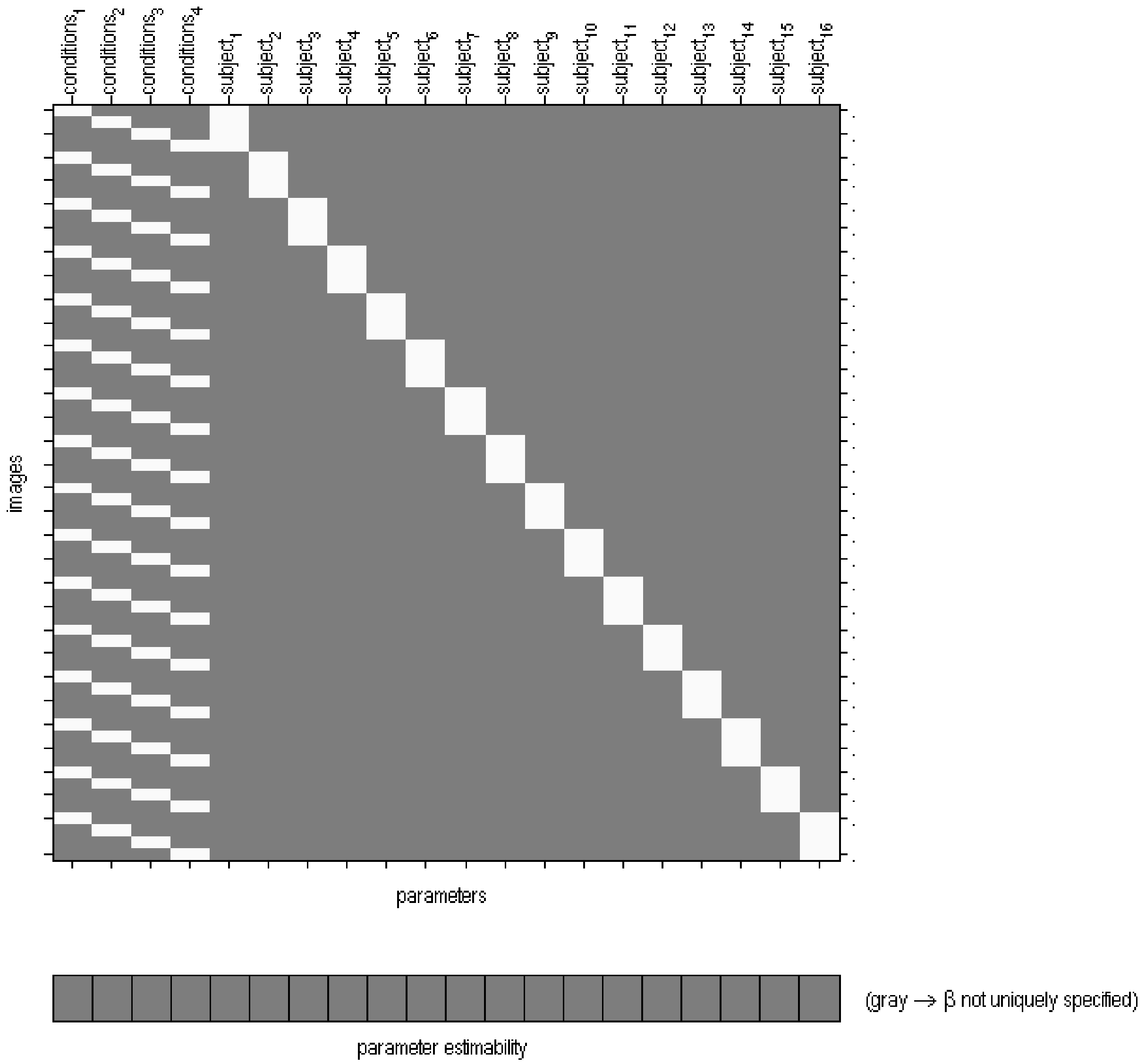

Design description...

Design : Flexible factorial
Global calculation : omit
Grand mean scaling : <no grand Mean scaling>
Global normalisation : <no global normalisation>
Parameters : 4 condition, +0 covariate, +16 block, +0 nuisance
20 total, having 19 degrees of freedom
leaving 45 degrees of freedom from 64 images

Figure S3
